# Mannans and endo-β-mannanases (MAN) in *Brachypodium distachyon*: expression profiling and possible role of the *BdMAN* genes during coleorhiza-limited seed germination

**DOI:** 10.1093/jxb/erv168

**Published:** 2015-04-28

**Authors:** Virginia González-Calle, Cristina Barrero-Sicilia, Pilar Carbonero, Raquel Iglesias-Fernández

**Affiliations:** Centro de Biotecnología y Genómica de Plantas (UPM-INIA), and ETSI Agrónomos, Campus de Montegancedo, Universidad Politécnica de Madrid, Pozuelo de Alarcón, 28223-Madrid, Spain

**Keywords:** *BdMAN* gene family, *Brachypodium distachyon*, coleorhiza, endo-β-mannanases, germination, *MAN* gene expression, mannan immunolocalization, mRNA *in situ* hybridization.

## Abstract

Immunolocalization of mannans in the seeds of *Brachypodium distachyon* reveals the presence of these polysaccharides in the root embryo and in the coleorhiza in the early stages of germination (12h), decreasing thereafter to the point of being hardly detected at 27h. Concurrently, the activity of endo-β-mannanases (MANs; EC 3.2.1.78) that catalyse the hydrolysis of β-1,4 bonds in mannan polymers, increases as germination progresses. The *MAN* gene family is represented by six members in the *Brachypodium* genome, and their expression has been explored in different organs and especially in germinating seeds. Transcripts of *BdMAN2*, *BdMAN4* and *BdMAN6* accumulate in embryos, with a maximum at 24–30h, and are detected in the coleorhiza and in the root by *in situ* hybridization analyses, before root protrusion (germination *sensu stricto*). *BdMAN4* is not only present in the embryo root and coleorhiza, but is abundant in the de-embryonated (endosperm) imbibed seeds, while *BdMAN2* and *BdMAN6* are faintly expressed in endosperm during post-germination (36–42h). *BdMAN4* and *BdMAN6* transcripts are detected in the aleurone layer. These data indicate that *BdMAN2, BdMAN4* and *BdMAN6* are important for germination *sensu stricto* and that *BdMAN4* and *BdMAN6* may also influence reserve mobilization. Whether the coleorhiza in monocots and the micropylar endosperm in eudicots have similar functions, is discussed.

## Highlight

Mannans present in the coleorhiza and root of *Brachypodium* seeds disappear early during germination *sensu-stricto*, while mannanase activity increases and three *BdMAN* transcripts are localized in these tissues.

## Introduction

Poaceae grains (caryopses) include the seed proper, formed by a triploid endosperm and a diploid embryo, surrounded by the maternal tissues of the seed coat (testa) and the pericarp. The coleorhiza is a non-vascularized multicellular embryonic tissue, covering the seminal roots of Poaceae seeds. The coleorhiza has been thought to have a role in protecting the emerging root (Sargent and Osborne, 1980) and, more recently, it has been also associated with the regulation of dormancy, since abscisic acid (ABA) sensitivity is reduced in this tissue during germination of non-dormant barley seeds and the gene encoding the HvABA8′OH-1 enzyme, that is critical for ABA degradation, is expressed in the coleorhiza. During germination, both in barley and in *Brachypodium* seeds, the coleorhiza is the first structure that protrudes after the pericarp and testa rupture (coleorhiza emergence), followed by the coleorhiza rupture that allows root emergence (root emergence), and indicates the end of germination *sensu stricto* (Millar *et al.*, 2006; Barrero *et al.*, 2009, 2012; Gao and Ayele, 2014).

The germination process can be separated into germination *sensu stricto* and subsequent reserve mobilization (post-germination) and has been more deeply investigated in eudicotyledonous than in monocotyledonous seeds. In *Arabidopsis thaliana*, *Sisymbrium officinale*, *Lepidium sativum* and *Nicotiana tabacum*, the germination *sensu stricto* occurs in two different steps; first, the testa ruptures and, afterwards, the micropylar endosperm breakage takes place, allowing the radicle to emerge (Leubner-Metzger and Meins, 2000; Nonogaki *et al.*, 2000; Müller *et al.*, 2006; Iglesias-Fernández and Matilla, 2010; Iglesias-Fernández *et al.*, 2011*a*, *b*; Weitbrecht *et al.*, 2011; Nonogaki, 2014). Addition of ABA to the imbibition medium specifically blocks endosperm weakening and prevents its rupture (Müller *et al.*, 2006; Piskurewicz *et al.*, 2009; Carrillo-Barral *et al.*, 2014). It is assumed that testa rupture is influenced by the driving force of the imbibed elongating radicle and that the endosperm rupture is mainly produced by the weakening of the endosperm cell walls (CWs) by enzymes, specifically those localized to the micropylar endosperm, such as endo-β-1,4-mannanases (MANs), endo-ß-1,3-glucanases, expansins, xyloglucan-transglycosylases/hydrolases (XTHs) and pectin-methylesterases (Leubner-Metzger, 2005; Nonogaki *et al.*, 2007; Iglesias-Fernández *et al.*, 2011*a*, *b*; Endo *et al.*, 2012; Martínez-Andújar *et al.*, 2012; Rodríguez-Gacio *et al*., 2012; Scheler *et al.*, 2014).

Since the endosperm CWs of several eudicot seeds are rich in mannans (Lee *et al.*, 2012), the MAN activity and the expression of *MAN* genes upon seed germination have been further characterized and their transcriptional regulation studied. In *A. thaliana*, four *MAN* genes (*AtMAN2*, *AtMAN5*, *AtMAN6* and *AtMAN7*) are expressed in germinating seeds and their transcripts are restricted to the micropylar endosperm and to the radicle, disappearing as soon as the radicle emerges. Moreover, knock-out mutants in the *AtMAN5*, *AtMAN6* and *AtMAN7* genes, as well as, in the *AtbZIP44* gene encoding an important activating transcription factor of *AtMAN7*, have a significantly retarded germination as compared to that of wild-type seeds, indicating a role for these *MAN* genes and their regulators during germination *sensu stricto* (Iglesias-Fernández *et al.*, 2011*a*, *b*, 2013; Rodríguez-Gacio *et al.*, 2012; Yan *et al.*, 2014).

*Brachypodium distachyon* is being considered a model species for the genetics and molecular genomics of cereals, due in part to its small sequenced genome (~355 Mbp), short life cycle, self-fertility, diploidy and its close phylogenetic relationship with important crop plants of the tribe Triticeae within the Poaceae family, such as wheat and barley (International Brachypodium Initiative, 2010; Mochida and Shinozaki, 2013; Girin *et al.*, 2014). In Poaceae seeds, the β-1,3-1,4-glucans are abundant in the endosperm cell walls (Burton and Fincher, 2009; Guillon *et al.*, 2011) and genes encoding hydrolytic enzymes involved in their degradation, such as endo-β-1,3-glucanases and endo-β-1,3-1,4-glucanases, have been associated with rice post-germination events (2–4 days of imbibition; Akiyama *et al*., 2004) and with the elongation of barley coleoptiles (Takeda *et al.*, 2010). Although mannan content is lower than glucan content in *Brachypodium* seeds (Rancour *et al.*, 2012), the function of mannans and MANs may be relevant in its germinating seeds.

In this work, mannan polysaccharides were immunolocalized to the root and the coleorhiza of germinating seeds early in imbibition, decreased thereafter at later stages, and the enzymatic activity of endo-β-mannanases increased as germination progressed. The *MAN* gene family of *B. distachyon* was annotated and the expression of its six members explored in vegetative and reproductive organs. Interestingly, genes *BdMAN2*, *BdMAN4* and *BdMAN6* were clearly induced upon seed germination and mRNA *in situ* hybridization analyses demonstrated that these transcripts were found in the coleorhiza and the root during germination *sensu stricto*. *BdMAN4* and *BdMAN6* were also expressed in the aleurone layer, and may also be involved in post-germinative reserve mobilization.

## Materials and Methods

### Biological material, growth conditions and germination assays

The diploid inbred *Brachypodium distachyon* strain Bd21 (kindly provided by Prof. Garvin from the University of Minnesota, USA; International Brachypodium Initiative, 2010) was used in this work. Seeds were surface-sterilized with 1% NaOCl for 10min and washed in sterile water, before germinating on Petri dishes, containing two filter papers (Whatman 3) moistened with 8ml of sterile water, at 22ºC in the dark for 2 d. They were then transferred to pots in the greenhouse under long-day conditions (16h/8h, light/darkness; light intensity 155 µmol photons m^−2^ s^−1^) for sampling roots (6-week-old plants), young and old leaves (6- and 12-week-old plants) and spikes. For the germination experiments that lasted up to 42h, seeds were incubated in the dark at 22ºC, in Petri dishes with moistened filter papers, using triplicate lots of 25 non-stratified after-ripened seeds (stored at 22ºC and 30% relative humidity in the dark for 3 months). Seed samples were separated into embryo and endosperm (de-embryonated seeds) at 0, 12, 24, 30, 36 and 42h of imbibition, and used for RNA quantification and for protein extraction to determine MAN enzymatic activity.

### Endo-β-1,4-mannanase (MAN) activity assays

Seed samples, obtained as described above, were homogenized in 100mM sodium acetate buffer (pH 4.5) containing 1M NaCl and 0.5% ascorbic acid, at 4ºC for 2h in an orbital shaker (VWR International Eurolab, Barcelona, Spain). The homogenates were centrifuged at 15,000 × *g* for 45min, and 80 μl of this supernatant was mixed with 150 μl of 0.25% mannan (1,4-β-D-mannan from carob; Megazyme International Ireland Ltd., Wicklow, Ireland). Incubation was at 30ºC for different periods of time and the enzymatic activity was determined by the increase in reducing sugar production per mg of protein, as determined by the 4-OH-Benzoic Acid Hydrazide (PAH-BAH; Sigma-Aldrich) method (Lever, 1977). The MAN from *Aspergillus niger* (Megazyme) was used to establish the control curve (one unit of MAN activity defined as the amount of enzyme that releases 1 nmol of reducing sugar per minute under the experimental conditions). Protein concentration was determined with the Bradford reagent (Bio-Rad Laboratories, Munich, Germany) using bovine serum albumin (BSA) as a standard.

### Bioinformatic tools: BdMANs identification and phylogenetic analysis

The deduced protein sequences of the six *MAN* genes were obtained from the *B. distachyon* genome using the TBLASTN tool at the Phytozome v8.0 Database (Goodstein *et al.*, 2012; www.phytozome.net), using the eight OsMAN proteins from the *Oryza sativa* genome as query sequences (Yuan *et al.*, 2007). The Interpro Program (PFAM database; Bateman *et al.*, 2002; http://pfam.sanger.ac.uk) was used to confirm the presence of the MAN conserved domain (glycosyl-hydrolase family 5). The complete amino acid sequences deduced from *B. distachyon*, O. sativa and *Arabidopsis thaliana MAN* genes were aligned by means of the CLUSTAL W program (Thompson *et al.*, 1994) and utilized to construct a phylogenetic dendrogram, using the neighbor-joining algorithm, a bootstrap analysis with 1,000 replicates, complete deletion and the Jones Taylor Thornton matrix, as settings. The MEME program software version 4.0 (Tamura *et al.*, 2007) was used to identify conserved motifs within the deduced MAN proteins and to validate the phylogenetic tree (Table 1). Default parameters were used with the following exceptions: the maximum number of motifs to find was set to 22 and the minimum width was set to eight amino-acid residues (Bailey *et al.*, 2009; http://meme.sdsc.edu/meme4_6_0/intro.htlm). A single capital letter represents a single residue relative frequency, if this is greater than 50% than twice that of the second most frequent residue in the same position. If no single residue matches these criteria, a pair of residues, represented by capital letters in brackets, is given if the sum of their relative frequencies exceeds 75%. If none of these characteristics are satisfied, a lowercase letter is given when the relative frequency of a residue is greater than 40%, if not, x is set.

**Table 1.** *Conserved amino acid motifs obtained by means of MEME (Bailey et al., 2009) from the analysis of the endo*-β-*mannanase proteins of* Brachypodium distachyon, Oryza sativa, and Arabidopsis thaliana The conserved amino acids, critical for enzyme activity, are in bold and the signature sequence for endo-β-mannanase enzymes is underlined.

**Motif**	**E value**	**Consensus sequences**
1	5.6e-1050	L[TS]S[DN]D[DS]FF[TS][DN]PT[IV][KR][DS][YF][YF] KN[HY]VK[AT]VLTR[VK]NT[LV]TG[VI]AY[KR]D[DE] PTI[FL] AWEL[MI]NEPRC*x*SDP[ST]GDTLQAW[IV] EEMAAYVKS[ILV]DP[NK]H
2	6.5e-718	DGGY[RN][AP]LQI[SA]PG[VR][YF][DN]ED[VM]F[QK] [GA]LDFV[IVL][AS]EA[RK][RK]HG[IV][RK]L[IL]L[SC] LVNN [WL][DE][DA][YF]GGK[AK]QYVRWA
3	1.40E-307	x[LY]GTDF[IV][AR]N[HNS]Q[VA]PGIDFA[ST][VI]H[SV] YP**D**xW[LF]P
4	7.90E-213	NGRPFY[VAI]NG[FW]N[AST]YWLMxxA[ASV]DP[AS]T
5	2.80E-202	[KRN]W[ML][DQ][AS]H[IV]ED[AG][AEQ][NA][IE]L[GKR] [KM]P[LVI]L[VFIL][TAG]**E**FG[KL]S
6	3.40E-199	WKxPG[YF][NST]T[AS]QRDA[FL][LFY]R[AT]VYD[KA] IYASAR[RK]GG[APS][GA][AV]G[AG]L[FV]**W**Q[LV]L
7	2.10E-172	[TS][AE][AMV][FL][RQ]QA[AS]A[MH]GL[TN]V[CA] RT**W**AF[SN]
8	2.20E-161	L[LV][ET][VI]GLEGFYGP[SG]SPER[KL]xVN
9	2.50E-146	GM[ED]x[YFM]DDG[YF][ESA][IV][VI][LF][AS]ES[PS] STAS[IL][IL]x[EN][HQ]S[RC]
10	2.90E-64	[PAG][EGS][DGW]G[FM]V[RE]R[NR]GT[QR][FL]V[LV]
11	8.20E-37	KDGKF[GD][NS][EG]FRE[DT]FM[KE]T[VI]Y[RN][IN] FLSSW[KE][EG]GVIGGGCLL**W**QLFP
12	4.40E-11	[YM][KHS][ILC]L[GC][FL][LAF][LSV][LC]LA[IVF][VI][YI] [LAF][SQ][FLSW]
13	4.40E-05	FNS[LR]C[AS]W[RS]CRWGC[KN]K
14	1.00E-02	[HL][KY][GK]EGDPGWQC[ST]IPP
15	2.60E+01	[CR]F[IV]SLSRSISSFI[QV][DQ]NF
16	3.70E+01	[HR]L[KL][DE][KQ]K[LS][IK]E[LM]CSHR[HP]
17	1.50E+02	QLA[AES]L[ND]G[QK][DF][AD][DE][AGV][LV][RC] RR[RA][RS][RS]
18	1.00E+03	[AG][AG][GP]GG[GW][LV]KLPVPWLQ
19	4.30E+04	KAFARA[ER][QR][AE][QR][PS]ARGKG
20	6.60E+04	[AI]VL[CH][ES][AS][SV][FY][IW][EI][LW][RT][CQ]NR
21	2.70E+05	SS[HP]RK[IT][GR][LS][GT]SGG[DS][SW]D
22	3.00E+05	L[GH][AH][GH][AR][AV]ALLVL[AL]CV[HV]

The major biochemical parameters of the deduced MAN proteins from *B. distachyon* and *O. sativa* are listed in Supplementary Table S1. Both isoeletric point (pI) and molecular weight (MW) were predicted using the Compute pI/MW tool (Gasteiger *et al.*, 2005; http://www.expasy.ch/tools/pi_tool.html) and the putative signal peptide cleavage site and sub-cellular localization were deduced by the SignalP 3.0 (http://www.cbs.dtu.dk/services/SignalP) and TargetP 1.1 tools (http://www.cbs.dtu.dk/services/TargetP/), respectively (Emanuelsson *et al.*, 2007).

### Real time quantitative PCR (RT-qPCR) analyses

Total RNA was purified from roots (6-week-old plants), young and old leaves (6- and 12–week-old plants) and spikes by the phenol/chloroform method (Lagrimini *et al.*, 1987). For the isolation of RNA from seeds at different stages of development (0–10 d after pollination: dap) and at different time points of germination (12, 24, 30, 36 and 42h), the protocol described by Oñate-Sánchez and Vicente-Carbajosa (2008) was followed. RNA samples were treated with DNAse I, RNAse-free (Roche Applied Science, Manheim, Germany) to avoid genomic DNA contamination. First-strand cDNA was synthesized with random hexamers using the High-Capacity cDNA Reverse Transcription Kit (Applied Biosystems, Foster City, CA, USA) according to the manusfacturer’s recommendations. Samples were stored at −20ºC until used.

PCR-amplification was performed in an Eco Real-Time PCR System (Illumina, San Diego, CA, USA). For each 10 µl reaction, 2 µl of DNA sample was mixed with 5 µl of FastStart SYBR Green Master (Roche Applied Science) and 0.25 µl of each primer (final concentration 500nM) plus sterile water up to final volume. Samples were subjected to thermal-cycling conditions of 95ºC for 10min and 40 cycles of 10 s at 95ºC and 30 s at 60ºC for annealing and extension, respectively. The melting curve was designed to increase from 55ºC to 95ºC, and the melting temperatures for each amplicon and primer efficiencies (Supplementary Table S2) were estimated using a calibration dilution curve and slope calculation (E=10^(−1/slope)^). The specific primers used are shown in Supplementary Table S2 and they were designed on the 3′-non-coding region using the Primer3Plus program (http://www.bioinformatics.nl/cgi-bin/primer3plus/primer3plus.cgi). The *BdGAPDH* gene (encoding glyceraldehyde 3-phospate dehydrogenase; Hong *et al.*, 2008) was used to normalize the data, since the expression of this gene was previously demonstrated to be constant throughout the period studied (Hernando-Amado *et al.*, 2012; González-Calle *et al*., 2014; Supplementary Fig. S1). Expression levels were calculated as the number of cycles needed for the amplification to reach a cycle threshold fixed in the exponential phase of the PCR (C_t_; Pfaffl, 2001). All analyses used three different biological replicates for each time-point and each one was made in triplicate. Means ± standard error (SE) of three independent experiments are indicated in the corresponding figures.

### Preparation of embedded material for microscopy

Samples were treated according to a modified version of the protocol described in Ferrandiz *et al.* (2000). After-ripened dry seeds and germinating seeds of *B. distachyon* (12h, 27h and 36h) were collected and, after removal of the lemma and palea, were infiltrated with the FAE solution (formaldehyde: acetic acid: ethanol: water, 3.5:5:50:41.5 by volume) for 40min in 25mm Hg vacuum; the seeds were then incubated at 4ºC for 3 d with gentle shaking. The samples were dehydrated through a graded series of aqueous ethanol mixtures and progressively embedded in paraffin after the replacement of ethanol with HistoClear (National Diagnostics, Hessle Hull, England). Thin sections of 8 µm were collected on glass slides and de-waxed.

### Heteromannan immunolocalization

The protocol used was a modification of those described in Marcus *et al.* (2010) and Guillon *et al.* (2011). In a pre-immunolabelling step, sections of embedded material as described above, were incubated in phosphate buffer sodium solution (PBS) and treated with 1mg/ml proteinase-K (Roche Applied Science). In order for the specific antibodies to have access to the heteromannans (mannans, glucomannans, and galactomannans) of the cell walls, β-1,3-1,4- glucans were removed by incubating the sections with a solution of 4 µg/ml lichenase [β-1,3-1,4- glucanase; Megazyme] for 2h at 37ºC, and then rinsed with de-ionized water. For heteromannan immunodetection, sections were first incubated at room temperature for 30min in a blocking solution (3% BSA, 1× PBS, and 5mM sodium azide; pH7), and then treated with primary anti-heteromannan antibody LM21 (PlantProbes, Leeds, UK) at a dilution of 1:5 in the same blocking solution but only containing 1% BSA for 2h. Sections were thoroughly washed in PBS containing 5mM sodium azide and then incubated for 2h in the same buffer containing the secondary rabbit antibody Anti-Rat IgG-FITC (Sigma-Aldrich) at a dilution of 1:100. The sections were extensively washed in PBS buffer and in water, mounted and examined in a confocal microscope (absorption 494nm; emission 521nm; Leica TCS-SP8, Leica, Wetzlar, Germany).

### mRNA *in situ* hybridization analyses

Pre-hybridization was carried out by incubating the sections in 0.2M HCl, neutralizing them and then treating them with 1mg/ml proteinase-K (Roche Applied Science). Samples were then dehydrated in an aqueous ethanol dilution series and hybridized with sense and anti-sense digoxigenin (DIG)-labelled RNA probes, corresponding to DNA fragments (200–300bp) derived from the 3′-non coding regions of the *BdMAN2*, *BdMAN4* and *BdMAN6* genes (Supplementary Table S3), synthesized with the DIG RNA labelling mix according to the manufacturer’s specifications (Roche Applied Science). Probes were hybridized at 52ºC overnight followed by two washes in 2× SSC (150mM NaCl, 15mM Na_3_-citrate) and 50% formamide for 90min at the same temperature. Incubation with the alkaline phosphatase-conjugated anti-digoxigenin antibody (Roche Applied Science) and colour detection was carried out according to the manufacturer’s instructions (Ferrandiz *et al.*, 2000). Sections were dried and examined on a Zeiss Axiophot Microscope (Carl Zeiss, Oberkochen, Germany), and images were captured and processed with the Leica Application Suite 2.8.1 build software (Leica).

### Protein and polysaccharide histological determinations

**Fig. 1.**
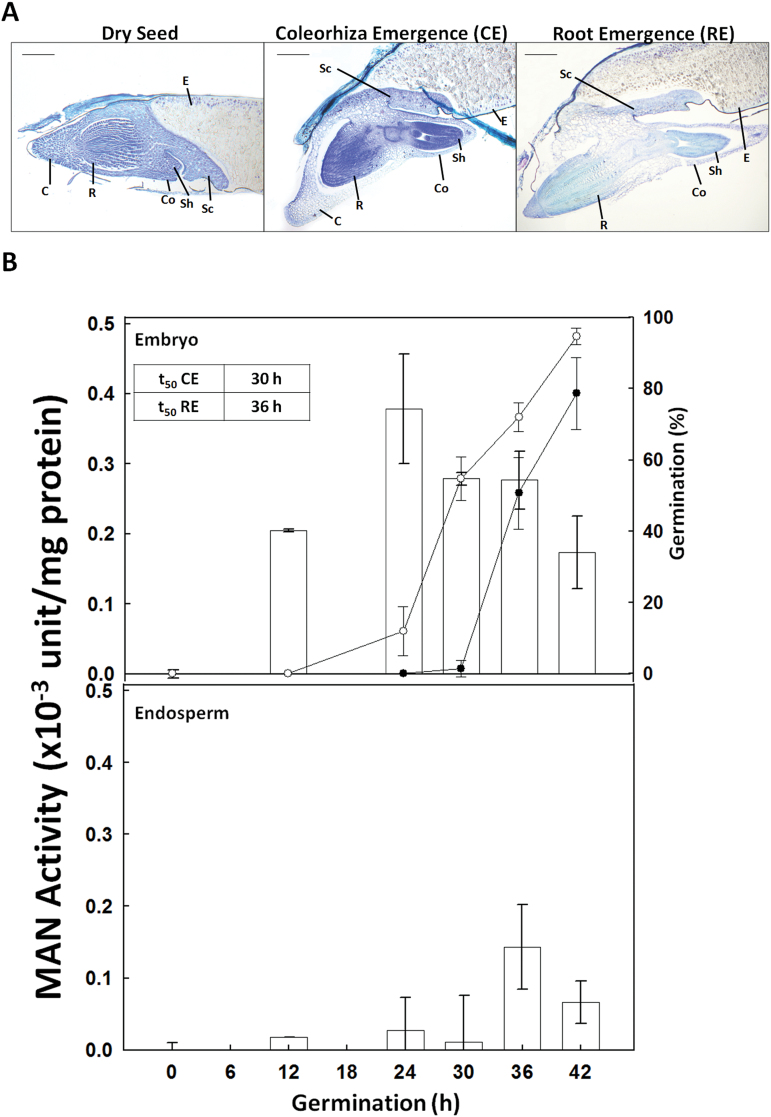
(A) Longitudinal sections of the different phases of *Brachypodium distachyon* germination *sensu stricto*, stained with toluidine blue. C, coleorhiza; Co, coleoptile; E, endosperm; Sc, scutellum; Sh, shoot; R, root. Scale bar, 200 μm. (B) Endo-β-mannanase activity (white bars) in embryo and endosperm (de-embryonated seed) upon *B. distachyon* seed germination (0–24h). One unit of MAN activity is defined as the amount of enzyme that releases 1 nmol of reducing sugar per minute and per mg of protein. Percentage germination evaluated as coleorhiza emergence (CE; open circles) and root emergence (RE; close circles) are represented. In the inset, the time needed for 50% of CE (t_50_CE) and RE (t_50_RE) is indicated. Data are means ± standard error (SE) of three technical replicates of three biological samples.

*B. distachyon* dry and germinating seeds were stained with 5% (w/v) toluidine blue (Merck, Darmstadt, Germany) for checking tissue integrity (Fig. 1A). Samples were stained with PAS reagent (0.5% w/v periodic acid-Schiff reagent) (Merck) to detect polysaccharides and with 1% (w/v) Naphthol Blue Black (Sigma-Aldrich) for proteins (Iglesias-Fernández and Matilla, 2010). Visualization was done on a Zeiss Axiophot Microscope (Carl Zeiss) and the images were captured and processed with the Leica Application Suite 2.8.1 build software (Leica).

## Results

### Enzymatic β-mannanase activity during *Brachypodium* seed germination

The time course of *sensu stricto* germination of *B. distachyon* seeds occurs in two different steps: first, the coleorhiza emerges (CE), and in a second step, the root emergence (RE) takes place (Fig. 1A). The enzymatic activity of MAN upon germination has been analysed separately in the embryo and in the de-embryonated seed (endosperm). As shown in Fig. 1B, dried seeds have no detectable MAN activity, but this progressively increases with germination, peaking at 24h in embryos, containing the coleorhiza (~0.4×10^–3^ units/mg protein), and decreasing to half this value at 42h (~0.2×10^–3^ units/mg protein). In endosperms, MAN activity is much lower than in embryos, and reaches its maximum level (~0.15×10^–3^ units/mg protein) at 36h of germination. Data from Fig. 1B indicate that MAN activity is maximum in embryos, just before reaching 50% of germination *sensu stricto* (t_50_CE=30h; t_50_RE=36h), suggesting that MAN is important for facilitating both coleorhiza and root emergence.

### Heteromannans are preferentially localized to the root tip and the coleorhiza in germinating seed embryos

Mannan polymers have been detected in longitudinal sections of *B. distachyon* germinating seeds (at 12 and 27h of imbibition) by *in situ* immunofluorescence labelling, using the LM21 antibody that specifically recognizes mannan polysaccharides (gluco- and galacto-mannans). To facilitate accession of the antibody to mannans in plant CWs, the seed sections have been previously treated with lichenase (β-1,3-1,4- glucanase; Marcus *et al.*, 2010).

**Fig. 2.**
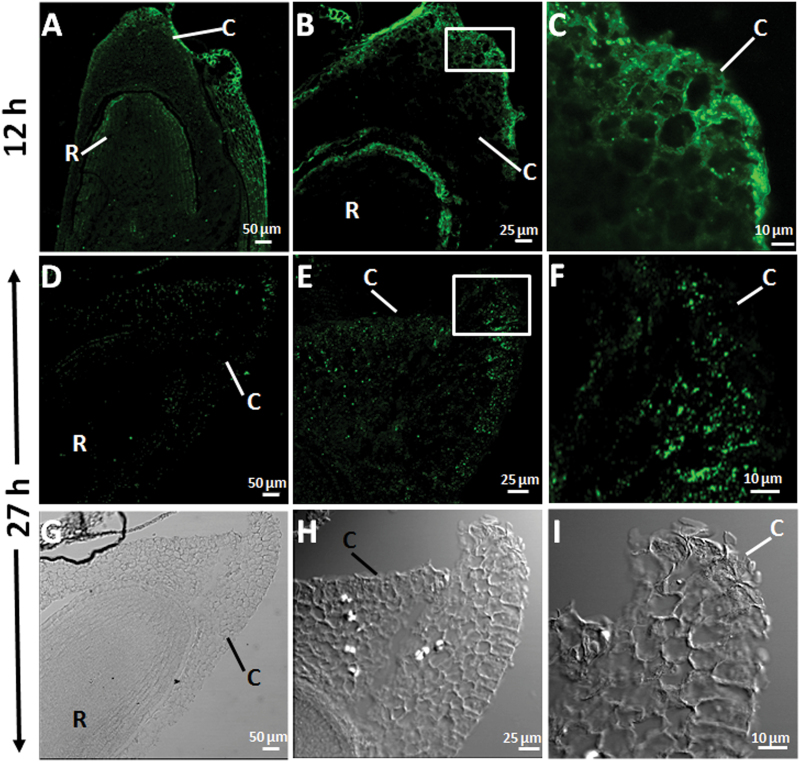
Mannan polymer immunolocalization at the root tip and the coleorhiza in longitudinal sections of *Brachypodium* germinating embryos at (A–C) 12h and at (D–F) 27h. (G–I) DIC images of D, E and F. (C, F, I) Close-up of the coleorhizae. C, coleorhiza; R, root. Scale bars: (A, D, G), 50 μm; (B, E, H), 25 μm; (C, F, I), 10 μm.

As shown in Fig. 2, at 12h of seed imbibition, seed mannan polymers are mainly localized to the periphery cells of the coleorhiza (C) and to the epidermis of the root tip (R) (Fig. 2A–C). Interestingly, these mannans are barely detected at later stages of germination (27h of imbibition; Fig. 2D–F). Differential interference contrast (DIC) images are shown in Fig. 2G–I. This observation together with data of MAN enzymatic activity (Fig. 1B) with a maximum at 24h in embryos, may suggest that the disappearance of the mannan polymers is due to the hydrolysis catalysed by endo-β-mannanases.

### The *Brachypodium* endo-β-mannanase gene family

**Fig. 3.**
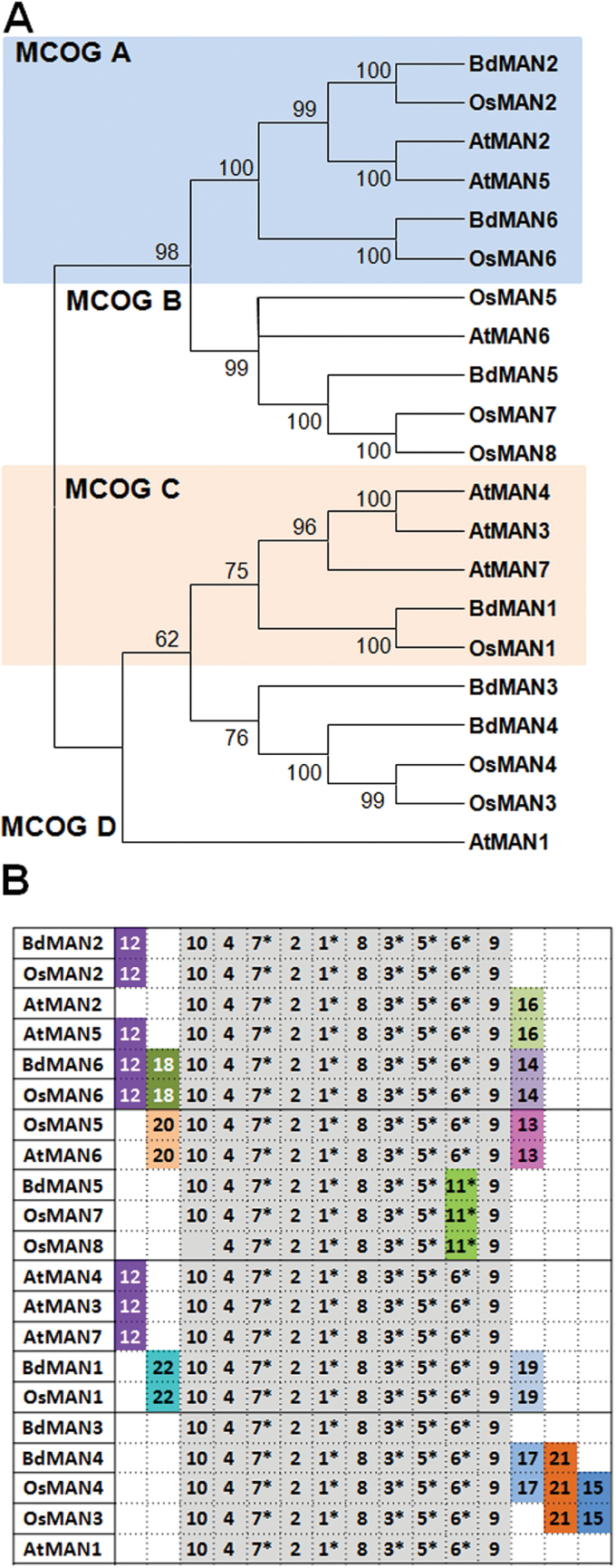
(A) Phylogenetic dendrogram with deduced protein sequence of the mannanase gene families form *Brachypodium distachyon*, *Oryza sativa* and *Arabidopsis thaliana*; bootstrapping values are indicated in the branches. (B) Schematic distribution of conserved motifs among the deduced protein sequences in the phylogenetic tree (A), identified by means of the MEME analysis. Asterisks indicate those motifs important for enzymatic activity. Motifs in grey share >85% of similar amino acid residues. This figure is available in colour at JXB online.

In order to get a deeper insight into the MAN function upon *B. distachyon* germination, it was decided to annotate and characterize further the BdMAN family. The already described MAN family from *O. sativa* (Yuan *et al.*, 2007) has been used to perform a TBLASTN against the whole *Brachypodium* genome (http://www.phytozome.net). Six predicted non-redundant MAN deduced proteins, with MW 43–52 KDa, and Ip 4.4–8.8, three of them with predicted signal peptides, have been identified and named according to their orthologues in rice (Supplementary Table S1). The MAN protein sequences from *A. thaliana* (AtMAN1-7) and *O. sativa* (OsMAN1-8) together with those from *B. distachyon* (BdMAN1-6) have been used to construct a phylogenetic unrooted tree by using the neighbor-joining algorithm. Four major clusters of orthologous groups (MCOGs) have been defined (A, B, C, D), supported by bootstrapping values higher than 62% (Fig. 3A) and by the occurrence of common motifs (Fig. 3B; MEME).

The search for conserved amino-acid motifs using the MEME software (http://meme.nbcr.net/meme/cgi-bin/meme.cgi) reveals that all MAN sequences have in common motifs described as critical for the enzymatic activity, such as 1, 3, 5, 6/11 and 7 (Fig. 3B, Table 1). The deduced signature sequence [AWEL(MI)NEPRC] of *Arabidopsis* and rice MANs (Yuan *et al.*, 2007), included in motif 1, is also present in *Brachypodium* MAN. Besides, members in MCOG A, BdMAN2, OsMAN2, BdMAN6 and OsMAN6 share motif 12, and BdMAN6 and OsMAN6 also share motifs14 and 18. In MCOG C, BdMAN1 shares with OsMAN1 motifs 19 and 22, and in MCOG D BdMAN4 shares motifs 17 and 21 with OsMAN4, but lacks motif 15 shared by the rice paralogues OsMAN3 and OsMAN4. Similarly, BdMAN5, OsMAN7 and OsMAN8 (MCOG B) have in common motif 11, but they do not share with OsMAN5 motifs 20 and 13. The MAN protein motifs from *A. thaliana* have been included for comparison (Iglesias-Fernández *et al.*, 2011*a*).

### Expression kinetics of selected *BdMAN* genes during seed maturation and germination

The expression pattern of the six *BdMAN* genes has been explored by RT-qPCR analysis in different organs: young (6 d) and old (12 d) leaves, roots (6 d) and spikes (mix of different stages; Supplementary Fig. S2). While *BdMAN1*, *2*, *3, 5* genes are not detected in leaves, *BdMAN4* gene expression in old leaves is ~10 times lower than in young leaves, and *BdMAN6* has the same low expression in young and old leaves. In roots, *BdMAN2*, *BdMAN4* and *BdMAN6* are expressed at low levels, and *BdMAN1*, *BdMAN2*, *BdMAN4* and *BdMAN6* transcripts are detected in spikes (Supplementary Fig. S2).

**Fig. 4.**
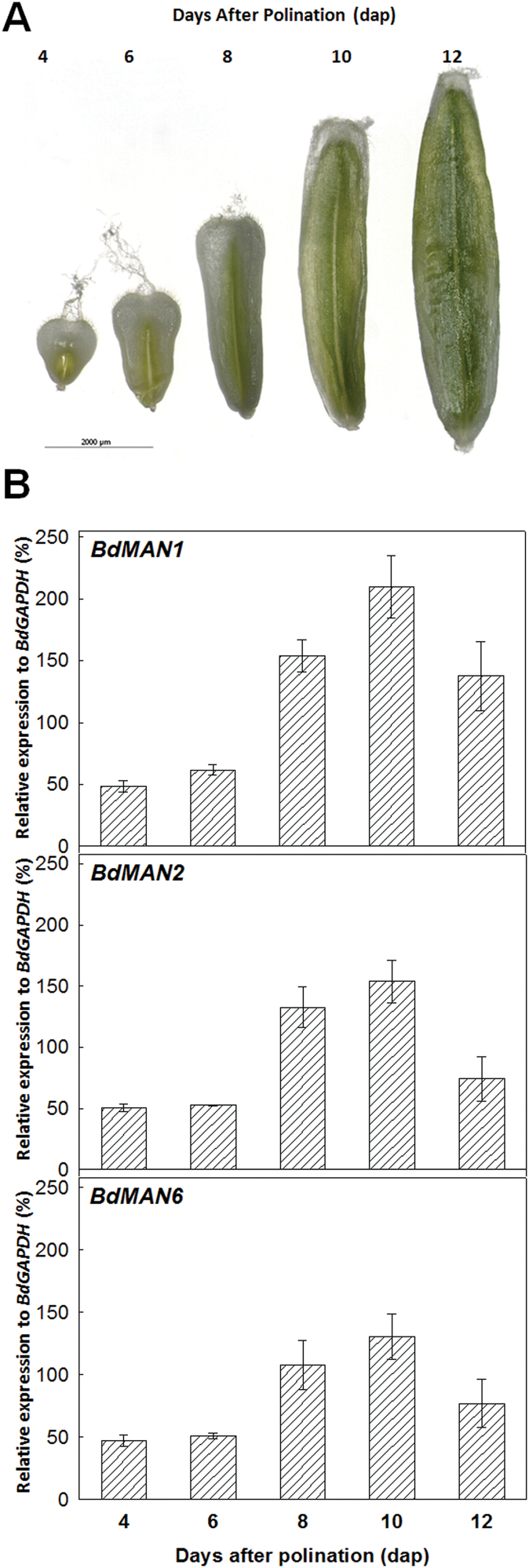
(A) Different stages of *Brachypodium distachyon* seed development (4, 6, 8, 10, 12 d after pollination; dap). (B) Expression of the *BdMAN1*, *BdMAN2* and *BdMAN6* genes by RT-qPCR during seed development. Data are means ± standard error (SE) of three technical replicates of three biological samples.

Since our preliminary data indicate that *BdMAN1*, *BdMAN2* and *BdMAN6* transcripts are abundant in developing seeds (see Supplementary Fig. S3A), the expression kinetics of these three genes has been established throughout seed maturation, at 4, 6, 8, 10 and 12 d after pollination (dap) (Fig. 4A). Although the gene *BdMAN1* is the most highly expressed during the late phases of seed development (8, 10, 12 dap), the expression patterns of *BdMAN1*, *BdMAN2* and *BdMAN6* show a progressive increase from 4 to 10 dap, reaching all of them their maximum expression at 10 dap when maturation is almost completed (Fig. 4B).

**Fig. 5.**
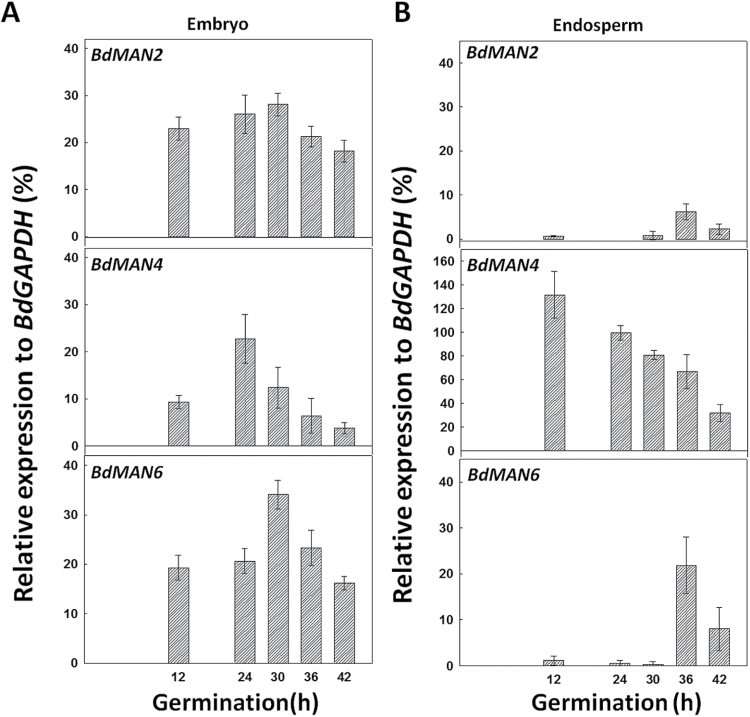
Transcript accumulation of *BdMAN2*, *BdMAN4* and *BdMAN6* in (A) embryos, and in (B) endosperms (de-embryonated seeds) upon *B. distachyon* seed germination. Data are means ± standard error (SE) of three technical replicates of three biological samples.

Data in Supplementary Fig. S3B indicate that genes *BdMAN2*, *BdMAN4* and *BdMAN6* are the most abundantly expressed ones during seed germination, and their expression kinetics has been more thoroughly analysed (Fig. 5). Germinating seeds taken at 12, 24, 30, 36 and 42h of imbibition, have been sectioned into embryos and de-embryonated seeds (endosperm). In germinating embryos, *BdMAN2*, *BdMAN4* and *BdMAN6* transcripts appear early upon imbibition (12h), before CE, and their maximum expression is attained between 24–30h (t_50_CE=30h) and it decreases as germination progresses (42h; Fig. 5A). However, the expression of *BdMAN4* in endosperms is high at early imbibition times (12h; ~140% relative to *BdGAPDH*), decreasing thereafter. The *BdMAN2* and *BdMAN6* transcripts have low expression in the endosperms of germinating seeds with a maximum at 36h of imbibition (<10 % for *BdMAN2* and ~25 % for *BdMAN6* relative to *BdGAPDH*, respectively), indicating a possible role in reserve mobilization for these *MAN* genes, and perhaps also for *BdMAN4* post-germination (Fig. 5B).

### *BdMAN2*, *BdMAN4* and *BdMAN6* transcripts are localized to different seed tissues during *B. distachyon* seed germination

**Fig. 6.**
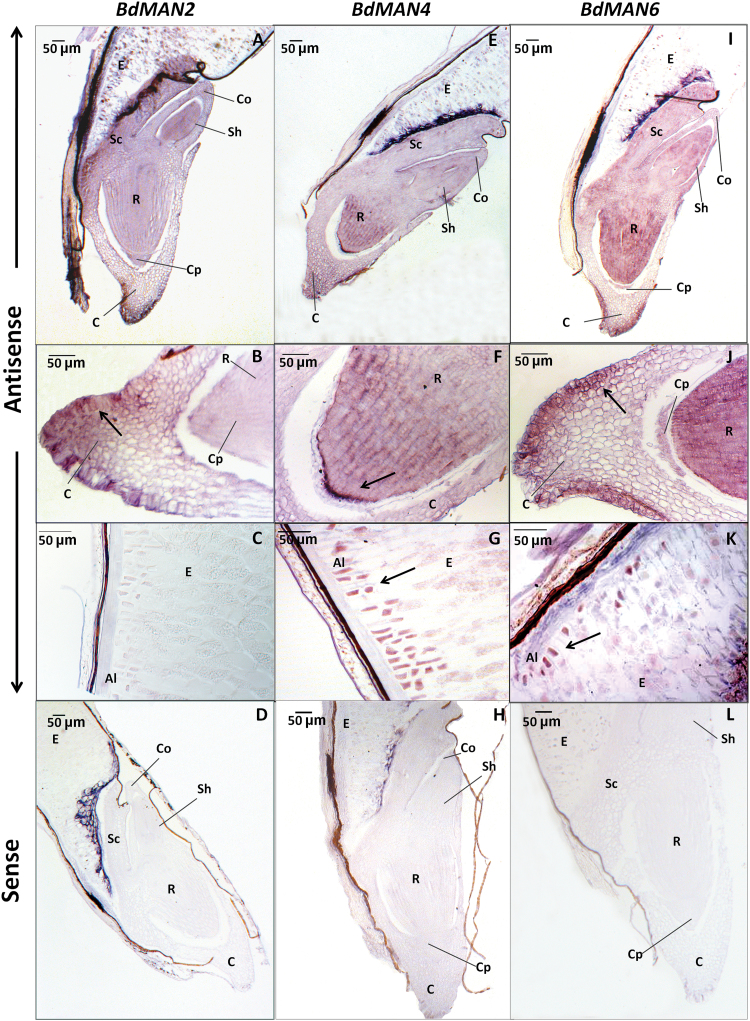
*In situ* mRNA hybridization analysis of *BdMAN2*, *BdMAN4* and *BdMAN6* in 27h germinating *Brachypodium* seeds. (A–D) *BdMAN2,* (E–H) *BdMAN4*, (I–L) *BdMAN6.* (A, E, I) Longitudinal sections of germinating embryos. (B, F, J) Close-up of the coleorhiza and the root tip. (C, G, K) Close-up of the endosperm and the aleurone. (D, H, L) Control sense probes. Al, aleurone layer; C, coleorhiza; Co, coleoptile; Cp, calyptra; E, endosperm; Sc, scutellum; Sh, shoot; R, root. The black arrow indicates the localization of transcripts. Scale bar, 50 μm.

To determine the spatial expression of *MAN* genes within the *B. distachyon* germinating seeds, *mRNA in situ* hybridization experiments have been done (Fig. 6). Longitudinal sections of seeds at 27h of imbibition have been hybridized to specific antisense and sense (as negative controls) probes for *BdMAN2, BdMAN4* and *BdMAN6.* The *BdMAN2* transcripts are mainly expressed in the periphery cells of the coleorhiza and are not detected in the aleurone layer (Fig. 6A–D). *BdMAN4* mRNA is localized preferentially to the tip and the apical meristem of the root, to the coleorhiza and to the aleurone layer (Fig. 6E–H), and the *BdMAN6* transcripts are detected not only at the coleorhiza, but also throughout the embryo and faintly also at the aleurone layer (Fig. 6I–L). *BdMAN6* transcripts are detected in the aleurone layer at 27h of imbibition by mRNA *in situ* hybridization, but they are scarcely detected in 24–30h germinating endosperms by RTqPCR (Fig. 5B), indicating that its expression could be diluted by the remaining endosperm during the RNA isolation process. As expected, no signal has been detected when sections have been hybridized with the corresponding sense probes for *BdMAN2*, *BdMAN4* and *BdMAN6* (negative controls; Figs 6D, 6H and 6L).

### Other histological observations of germinating *B. distachyon* seeds

**Fig. 7.**
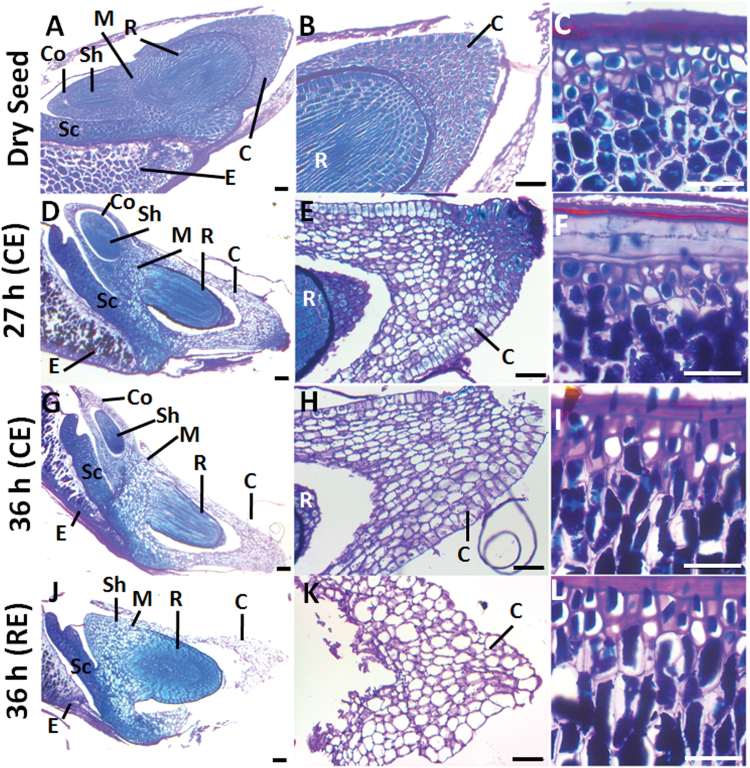
Polysaccharide and protein mobilization upon *B. distachyon* seed germination. Bright field microscopy of longitudinal seed sections stained with PAS-Naphthol Blue Black. (A, D, G, J) Longitudinal sections from dry and water-imbibed seeds at 27h and 36h. (B, E, H, K) Close-up of the coleorhiza in A, D, G, J and (C, F, I, L) close-up of the endosperm in A, D, G, J, respectively. Proteins stain in blue and polysaccharide-rich cell walls in pink. C, coleorhiza; Co, coleoptile; E, endosperm; M, mesocotile; Sc, scutellum; Sh, shoot; R, root. Scale bar: 50 μm.

Longitudinal sections of *B. distachyon* seeds (dry and water imbibed at 27 and 36h; including seeds before and after root emergence) have been stained with PAS reagent to detect insoluble polysaccharides (mainly cellulose and starch) and with Naphthol Blue Black for proteins. As shown in Fig. 7A–C, dry seeds have abundant protein bodies (PBs; blue stained) and thick cell walls (CWs; pink stained) in the root, coleorhiza and endosperm cells. When the coleorhiza emerges (27h of imbibition) the PBs in the coleorhiza (C) and in the mesocotyl (M) cells start to hydrolyze, while the endosperm cells are full of reserves at this stage (Fig. 7D–F). After 36h, when the seed coat ruptures but before root emergence (RE), the coleorhiza cells start to elongate and its protein bodies (PBs), as well as, those of the mesocotyl (M) are almost completely consumed, while those of the coleoptile (Co) initiate their degradation (Fig. 7G–I) and those of the endosperm remain as in the dry seeds (Fig. 7C, 7I). Finally, when root emergence (RE) takes place, the lateral part of the coleorhiza breaks (≥36h of imbibition) and its PBs are fully degraded, while those in the endosperm cells are almost intact (Fig. 7J–L).

## Discussion

In this work, mannans and endo-β-mannanases (MAN) in *Brachypodium distachyon* have been investigated in order to establish whether they are important in the germination of these monocotyledonous seeds. Mannans have been immunolocalized in the embryo root and the coleorhiza in the early stages of germination and these polymers decrease upon imbibition while the enzymatic activity of MAN increases. The *MAN* gene family in *B. distachyon* has been annotated and the gene expression of the six members of this family has been explored in different vegetative and reproductive organs, and, more specifically, in germinating seeds. Three of these genes, *BdMAN2*, *BdMAN4* and *BdMAN6*, are highly induced in germinating embryos and their transcripts are localized to the coleorhiza and the root, and *BdMAN4* and *BdMAN6* appear also in the aleurone layer. These facts indicate that the BdMAN enzymes should be spatially distributed in the seed in the vicinity of their putative substrates, thus contributing to the mannan hydrolysis and to the loosening of the coleorhiza cell walls, thereby facilitating root protrusion (germination *sensu stricto*).

During seed development, *BdMAN1*, *BdMAN2* and *BdMAN6* genes are expressed, and their mRNAs are abundant at the middle and late maturation stages. Upon cereal seed maturation, several tissues undergo a progressive enlargement of their cells, a process that involves nutrient remobilization and CW softening to allow cell expansion; to this aim, the participation of a complex set of hydrolytic enzymes have been described (Domínguez and Cejudo, 2014). These data indicate that the BdMAN1, BdMAN2 and BdMAN6 proteins could contribute to such a process during *Brachypodium* seed development.

The enzymatic analysis in the embryos of germinating seeds shows a maximum of MAN activity at 24h of imbibition, just before the coleorhiza emergence (CE_50_=30h), and this enzymatic activity progressively decreases to 50% at 42h. However, MAN activity in de-embryonated seeds (endosperms) is low, with a maximum at 36h of imbibition, when germination *sensu stricto* is almost completed [~100% coleorhiza emergence (CE); ~80% root emergence (RE) at 42 h]. These data point out to a more important role of the MAN activity in the embryo than in the endosperm during germination *sensu stricto*. In rice, MAN activity and expression of the *OsMAN1*, *OsMAN2* and *OsMAN6* genes have been detected in the aleurone layer only after 48h of imbibition when 100% of germination has been achieved. This MAN activity is associated with reserve mobilization, a clear post-germinative event (Ren *et al.*, 2008). In barley, the HvMAN1 enzyme has been purified from 10-day-old seedlings and its catalytic parameters established, although its physiological role has not been investigated (Hrmova *et al.*, 2006).

The function of the coleorhiza tissue in the grasses has been classically associated to a protective function of the growing root during germination, but other physiological functions are being uncovered, such as our observations of the hydrolysis of proteins (disappearance of PBs) or the decrease in mannan content detected within the coleorhiza cells during *Brachypodium* germination *sensu stricto*. Nowadays, and similarly to what has been proposed for the endosperm of eudicot seeds (Piskurewicz *et al.*, 2009), the coleorhiza is being considered to be a key tissue preventing root emergence in dormant barley seeds (Millar *et al.*, 2006; Barrero *et al.*, 2009). These authors have hypothesized that root emergence may not depend only on the softening of the coleorhiza, driven by CW remodelling enzymes, but also by the expansive force of the imbibing root cells. Important transcriptional changes in the barley coleorhiza associated to the dormancy degree have been found and these differences affect mainly the expression of CW modifying genes (mannanases among them), nitrate and nitrite reductase genes etc. (Barrero *et al.*, 2009). The cytosolic nitrate reductase is an important source of the hormone nitric oxide (NO) that is involved in promoting seed germination (Arc *et al.*, 2013). Therefore, the coexistence at the coleorhiza of NO and mannanases, and perharps proteases of the CatepsinB3 type (Iglesias-Fernández *et al.*, 2014), should have an influence in the seed germination of the grasses.

The *Arabidopsis* radicle tip has been described as the primary location of growth-promoting genes and its surrounding-cells the centre for CW expansion (Bassel *et al.*, 2014). Moreover, a dual enzymatic activity for MAN (hydrolase and transglycosylase activities) has been described; the transglycosylase activity being more related to cell expansion, as occurs in the radicle before protrusion, and the hydrolytic activity could be relevant for weakening of the CWs of the embryo-surrounding tissues (Schröder *et al.*, 2009; Iglesias-Fernández *et al.*, 2011*a*, *b*). It is remarkable that the *BdMAN4* transcripts are localized to the aleurone layer during imbibition (27h), when practically no MAN activity is detected in the de-embryonated (endosperm) seed, suggesting a possible accumulation of these transcripts and their corresponding proteins as inactive forms in the aleurone cells. Interestingly, the BdMAN4 deduced protein sequence has a predicted signal peptide for the secretory pathway and it is possible that the BdMAN4 isozyme could be transported later on during post-germinative reserve mobilization from the aleurone to the endosperm cells through the apoplastic space. In *Arabidopsis* the AtMAN7 and in poplar the PtrMAN6 proteins also contain signal peptides and have been localized to the apoplast, indicating that the mature MANs could be mobilized to the outer space (Iglesias-Fernández *et al.*, 2013; Zhao *et al.*, 2013).

Several authors have proposed that polysaccharides (β-1,3-1,4-glucans, mannans and others) present at the endosperm CWs of the Poaceae grains, not only have a structural function, but also a storage role (Guillón *et al.*, 2012). Heteromannans, although globally less abundant in these seeds than the β-glucans, are concentrated not only in the coleorhiza and in the root but also these polymers are found in the aleurone layer and storage endosperm of *B. distachyon* (Guillón *et al*., 2011). In this context, our data demonstrate that MAN activity is important for the weakening of the coleorhiza cell walls and for the expansion of the root cells, thus facilitating germination *sensu stricto*, but it may be also important for the mannan hydrolysis in endosperm during post-germinative reserve mobilization.

## Supplementary data

Supplementary data are available at *JXB* online.

Supplementary Fig. S1. Transcription levels of the housekeeping (*BdGAPDH* gene), presented as Ct mean values, in different organs, in developing seeds and during seed germination of *B. distachyon*.

Supplementary Fig. S2. Transcripts analysis by RTqPCR of the *BdMAN1-6* genes in different organs.

Supplementary Fig. S3. Expression analysis by RT-qPCR of *BdMAN1-6* genes in developing seeds and germinating seeds.

Supplementary Table S1. Major biochemical characteristics of *Brachypodium distachyon* and *Oryza sativa* endo-β-mannanase proteins.

Supplementary Table S2. Oligonucleotide sequences, amplicon length and PCR efficiency of the primers used for RT-qPCR analyses.

Supplementary Table S3. Primers used for the synthesis of the *in situ* mRNA hybridization probes.

Supplementary Data
