# Supplementary material for: Mannans and endo-β-mannanases (MAN) in Brachypodium distachyon: expression profiling and possible role of the BdMAN genes during coleorhiza-limited seed germination
Source: J Exp Bot. 2015 Apr 28;66(13):3753–64. doi: 10.1093/jxb/erv168 (PMC4473977; doi:10.1093/jxb/erv168)
Supplement: Supplementary Data [file supp_66_13_3753__index.html]

Mannans and endo-β-mannanases (MAN) in Brachypodium distachyon: expression profiling and possible role of the BdMAN genes during coleorhiza-limited seed germination — Mannans and endo-β-mannanases (MAN) in Brachypodium distachyon: expression profiling and possible role of the BdMAN genes during coleorhiza-limited seed germination — Supplementary Data 

# Mannans and endo-β-mannanases (MAN) in *Brachypodium distachyon*: expression profiling and possible role of the *BdMAN* genes during coleorhiza-limited seed germination

## Supplementary Data

Data files

**Files in this Data Supplement:**

- Supplementary Data - Supplementary Data
