# Supplementary material for: Mannans and endo-β-mannanases (MAN) in Brachypodium distachyon: expression profiling and possible role of the BdMAN genes during coleorhiza-limited seed germination
Source: J Exp Bot. 2015 Apr 28;66(13):3753–64. doi: 10.1093/jxb/erv168 (PMC4473977; doi:10.1093/jxb/erv168)
Supplement: Supplementary Data [file supp_erv168_jexbot145110_file001.pdf]

Mannans and endo-β-mannanases (MAN) in *Brachypodium distachyon*: Expression profiling and possible role of the *BdMAN* genes during coleorhiza-limited seed germination

Virginia González-Calle, Cristina Barrero-Sicilia[1], Pilar Carbonero, Raquel Iglesias-Fernández*

Centro de Biotecnología y Genómica de Plantas (UPM-INIA), and E.T.S.I. Agrónomos, Campus de Montegancedo, Universidad Politécnica de Madrid, Pozuelo de Alarcón, 28223-Madrid, Spain

Supplemetary data:

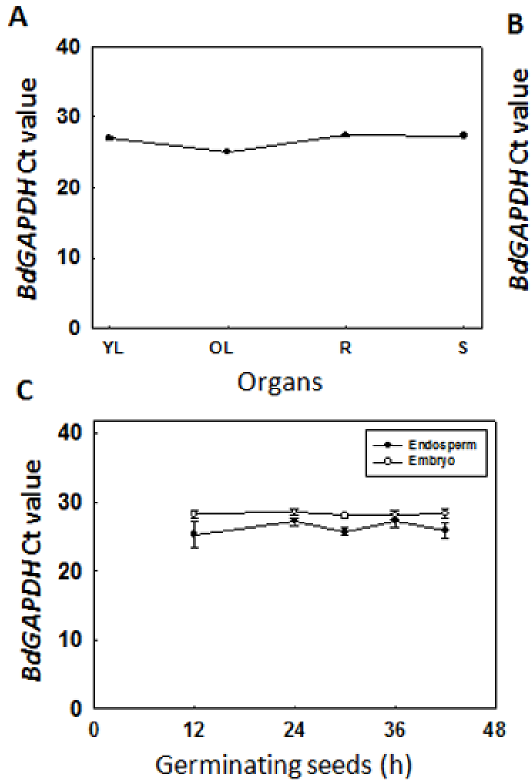

Supplementary Fig. S1. Transcription levels of the housekeeping (*BdGAPDH* gene), presented as Ct mean values, in different organs (OL: Old Leaves; R: Roots; S: Spikes; YL: Young Leaves**; A)**, in developing seeds (dap: days after pollination**; B)** and during seed germination **(**form 12 to 42 h; **C)** of *B. distachyon*.

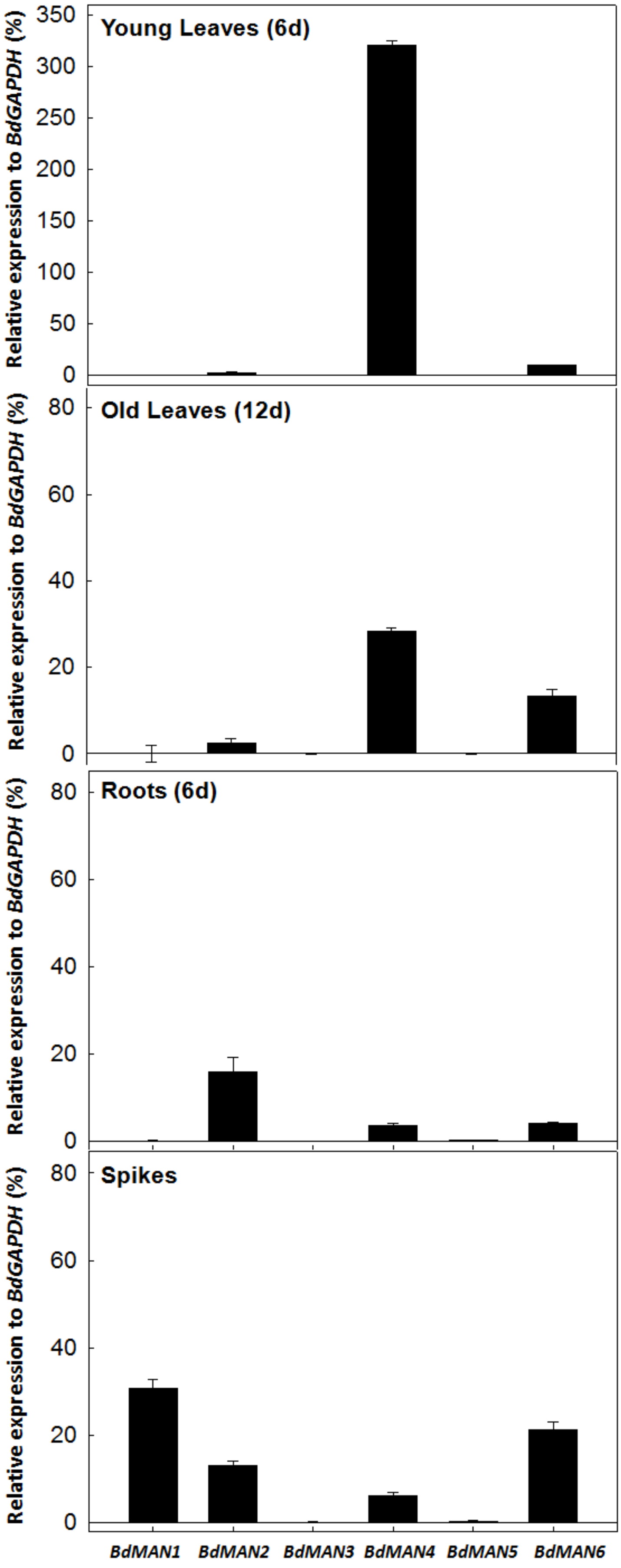

Supplementary Fig. S2. Transcripts analysis by RTqPCR of the *BdMAN1-6* genes in different organs. Young leaves (6 d); Old leaves (12 d); Roots (6 d) and Spikes. Data are means ± standard error (SE) of three technical replicates of three biological samples.

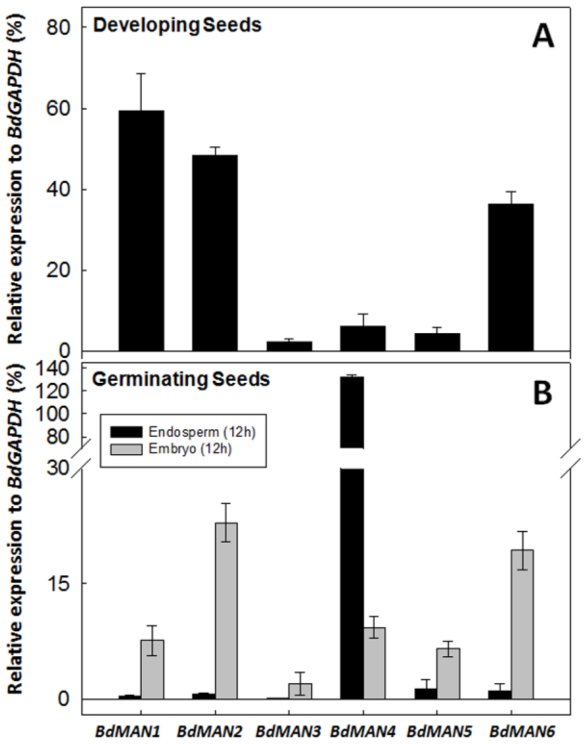

Supplementary Fig. S3. Expression analysis by RT-qPCR of *BdMAN1-6* genes in developing seeds (mix of different stages; **A)** and germinating seeds (12 h of imbibition; **B)**. Embryos and de-embryonated seeds (endosperm) were separately analysed of *Brachypodium distachyon*. Data are means ± standard error (SE) of three technical replicates of three biological samples.

Table S1. Major biochemical characteristics of *Brachypodium distachyon* and *Oryza sativa* endo-β-mannanase proteins. SL: Sub-cellular localization

Protein name	Locus	Protein size (Aa)	pI	Mw(Da)	Signal peptide position	SL prediction
**BdMAN1**	Bd2g45790	417	8.60	51765.15	Cleavage site 21-22	Secretory pathway (P=0.78)
**BdMAN2**	Bd2g49682	446	6.24	49945.77	No	Secretory pathway (P=0.92)
**BdMAN3**	Bd3g57290	413	6.84	45112.05	Cleavage site 30-31	Secretory pathway (P=0.88)
**BdMAN4**	Bd1g32107	468	5.67	51765.15	Cleavage site 20-21	Secretory pathway (P=0.85)
**BdMAN5**	Bd4g44340	389	4.44	43266.27	No	Other (P=0.91)
**BdMAN6**	Bd1g42770	436	8.75	49158.23	No	
**OsMAN1**	Os01g0663300.1	432	9.27	46874.91	Cleavage site 30-31	Secretory pathway (P= 0.96)
**OsMAN2**	Os01g0746700.1	445	5.79	50062.80	No	Secretory pathway (P= 0.90)
**OsMAN3**	Os03g0828300.1	468	6.02	51216.68	Cleavage site 23-24	Secretory pathway (P= 0.97)
**OsMAN4**	Os03g0828500.1	461	5.38	50739.43	No	------------
**OsMAN5**	Os05g0319100.1	491	5.60	53465.08	No	Secretory pathway (P=0.70)
**OsMAN6**	Os06g0311600.1	440	8.12	49455.08	No	Secretory pathway (P=0.96)
**OsMAN7**	Os11g0118200.1	379	4.95	42984.55	No	------------
**OsMAN8**	Os12g0117300.1	372	4.85	41839.18	No	Secretory pathway (P=0.96)

Table S2. Oligonucleotide sequences, amplicon length and PCR efficiency of primers used for RT-qPCR analyses.

Gene	Primer sense	Primer antisense	Amplicon size (bp)	Tm (^{0}C)	Slope	E*
BdGAPDH	CTCCCGCTATTTCGTTTGTC	TGAAGATGTTGGAGCTGACG	76	71.5	-3.07	2.11
BdMAN1	TATTCATCCGGGAGAGAAGG	ATTGGCATCTCCTGATCCAC	104	71.2	-3.36	1.98
BdMAN2	GGTGTACGACATCGCCTACG	GAGAAGCCGTCGTGGTAGTC	108	83.3	-4.22	1.73
BdMAN3	TGTATGACGCGATCTACGC	ATCACCTGCCAGAACATTCC	70	77.2	-3.64	1.88
BdMAN4	TAGGTTCAGTTTCAGGTTG	ACACTTGCACTGCACAT	120	70.3	-3.09	2.10
BdMAN5	CCAAGATCCAATCGTGAAATG	CTCACAGAAGCATAAGACCCAAC	86	65,9	-3.16	2.07
BdMAN6	GCTCATCAAAGTTGCCTGTG	TTACCGGCGCAACTAGAAAG	106	71,7	-3.40	1.96

*E=efficiency=$10^{(-1/slope)}$

Table S3. Primers used for the synthesis of the *in situ* mRNA hybridization probes

**Primer name**	**Sequence 5'-3'**
BdMAN2-insitu-s	CTCTCTCAAGCCGAAGCACC
BdMAN2-insitu-as	CGTAGGCGATGTCGTACACC
BdMAN4-insitu-s	CTGCCGACTCGTTTCGTCTC
BdMAN4-insitu-as	GACCAATCATCCTGACCGAC
BdMAN6-insitu-s	GCAAATAAAGCCCAAACTAC
BdMAN6-insitu-as	GTCCGCCAAGTTTATACAAC

s, sense; *as*, antisense
